# Isolated Optic Neuritis as the Presenting Feature of Neurosarcoidosis: A Case Report and Diagnostic Approach

**DOI:** 10.7759/cureus.96172

**Published:** 2025-11-05

**Authors:** Ambuj Bhalla, Zeeshan Zubair, Uzair Hamid, Jorge Kattah

**Affiliations:** 1 Neurology, University of Illinois College of Medicine Peoria, Peoria, USA

**Keywords:** methotrexate, neurosarcoidosis, noncaseating granulomas, optic nerve enhancement, optic neuritis, vision loss

## Abstract

We report a rare case of neurosarcoidosis presenting as isolated optic neuritis in a 43-year-old man with a history of obstructive sleep apnea and daily marijuana use. The patient experienced progressive, painless unilateral vision loss over six weeks, highlighting an atypical initial manifestation of this condition.

Ophthalmologic examination revealed left optic disc swelling, afferent pupillary defect, and impaired color vision. MRI demonstrated left optic nerve enhancement, while chest imaging identified bilateral hilar lymphadenopathy with noncalcified nodules. Mediastinoscopic biopsy confirmed noncaseating granulomas, establishing a diagnosis of sarcoidosis.

Treatment with high-dose corticosteroids followed by methotrexate resulted in improved visual acuity from counting fingers to 20/40, with resolution of optic nerve enhancement observed at one-year follow-up. This case underscores the critical role of early recognition and timely immunosuppressive therapy in preventing irreversible vision loss in neurosarcoidosis.

## Introduction

Sarcoidosis is a chronic, multisystemic inflammatory disorder characterized by the presence of noncaseating granulomas in affected tissues. Although the exact etiology remains unknown, it is believed to arise from an exaggerated immune response to environmental or infectious antigens in genetically predisposed individuals [1]. The disease most commonly affects adults between the ages of 20 and 40 and has a higher prevalence among African American populations and women [2].

Over 90% of sarcoidosis cases involve the intrathoracic organs, most notably the lungs and intrathoracic lymph nodes, often presenting with bilateral hilar lymphadenopathy, pulmonary infiltrates, or diffuse micronodular opacities [3]. Common systemic symptoms include fatigue, fever, night sweats, and weight loss, while specific organ involvement may manifest as cough and dyspnea (pulmonary), erythema nodosum (cutaneous), or uveitis (ocular) [4].

Neurosarcoidosis, a rare yet potentially disabling manifestation of sarcoidosis, occurs in approximately 5%-15% of cases [5]. It can affect any part of the nervous system, including the cranial nerves, brain parenchyma, meninges, spinal cord, and peripheral nerves [6]. Optic nerve involvement/neuritis, while rare and present in approximately 1%-5% of patients with neurosarcoidosis, can lead to severe and often irreversible visual loss if not recognized and treated promptly [7,8]. While typically painful in ~90% of cases, optic neuritis does demonstrate painless presentation in approximately 10% of patients and is well documented [7].

In this report, we describe a rare case of isolated optic neuritis (no initial systemic symptoms) as the sole neurological manifestation of biopsy-confirmed sarcoidosis, resulting in significant vision loss. By sharing this case, we aim to raise awareness of atypical and rare presentations of sarcoidosis, emphasize the importance of early recognition and diagnosis, and highlight the need for timely intervention to prevent long-term complications.

## Case presentation

A 43-year-old man with obstructive sleep apnea, obesity, osteoarthritis, hydronephrosis, and degenerative disc disease, who smokes marijuana daily and has mild cough and wheezing at baseline, presented to the emergency department (ED) (Day 0) after six weeks of progressive, painless vision loss in his left eye. He initially experienced intermittent blurring and a “black film” over his visual field that gradually worsened until he could perceive only shapes and shadows. He denied any headaches, systemic symptoms, or other neurological complaints.

Initial evaluation

An outpatient ophthalmology evaluation three weeks earlier documented left optic disc swelling on fundoscopy, constricted visual fields, reduced color vision (2/8 Ishihara plates), and an afferent pupillary defect, prompting neuroimaging. On presentation to the emergency department, his right eye visual acuity remained at baseline myopia, while the left eye measured 20/70, later worsening to light perception and counting fingers. Fundoscopy confirmed grade 2-3 unilateral papillitis, red desaturation, and complete loss of color plate recognition.

**Figure 1:**
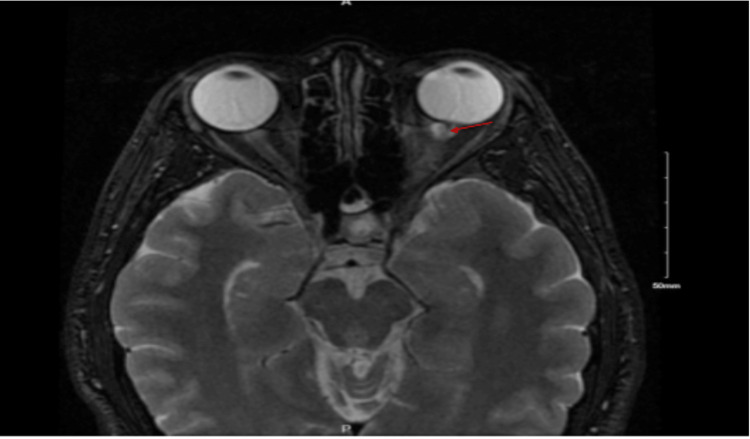
T1 fat-saturated MRI of the orbits demonstrating multifocal enhancement of the left optic nerve (red arrow) during acute presentation, consistent with optic neuritis.

Concerns for optic neuritis prompted referral for neuroimaging. An MRI of the brain and orbits was obtained on ED presentation, revealing multifocal contrast enhancement of the left optic nerve without T2 hyperintensity or surrounding edema (Figure 1), raising a differential diagnosis of neoplasm versus inflammatory or granulomatous process.

Given the patient’s mild periorbital discomfort with eye movements and clinical picture, on Day 2, he was started on high-dose intravenous methylprednisolone (1 g daily) for five days with a subsequent 30-day oral corticosteroid taper.

Cerebrospinal fluid studies and an extensive laboratory workup were also obtained to further elucidate the etiology of this patient’s presentation, including cell count, cytology, viral and bacterial PCR panels (cytomegalovirus (CMV), *Bartonella*, Lyme, and herpes), NMO/AQP4 antibodies, and kappa free light chains. Special stains for fungus, acid-fast bacilli, and flow cytometry/molecular studies to exclude lymphoma were also obtained. These were all negative, prompting a further workup for etiology determination.

Pulmonological workup and biopsy

**Figure 2:**
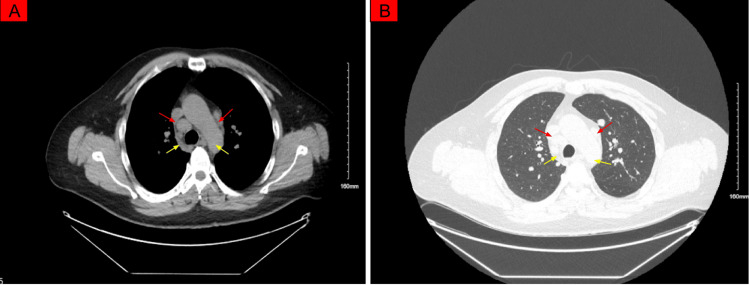
A: Axial CT scan showing bilateral hilar lymphadenopathy (red arrows) and mediastinal nodes (yellow arrows). B: Coronal CT scan highlighting noncalcified pulmonary nodules (red arrows) and hilar lymphadenopathy (yellow arrows).

To evaluate for systemic causes of optic neuritis, a screening chest radiograph was obtained on Day 24, revealing prominent central pulmonary vasculature. This finding prompted a noncontrast chest CT, which identified bulky mediastinal and bilateral hilar lymphadenopathy with multiple noncalcified pulmonary nodules (largest measuring 12×11 mm) (Figure 2A, 2B). Laboratory studies at this time showed normal serum angiotensin-converting enzyme (ACE), positive atypical perinuclear antineutrophil cytoplasmic antibody (pANCA), and elevated lactate dehydrogenase.

**Figure 3:**
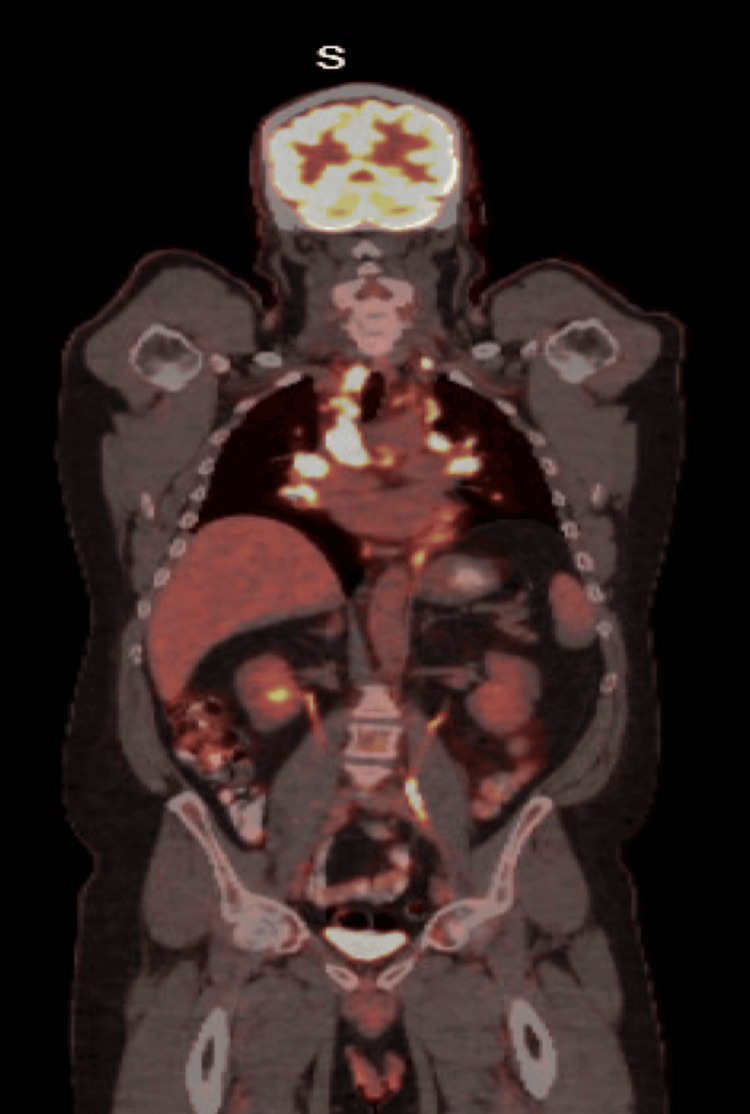
PET-CT scan highlighting FDG-avid supraclavicular, mediastinal, hilar, and pulmonary lymph nodes. FDG: fluorodeoxyglucose

Given the CT abnormalities, PET-CT was performed on Day 30, showing extensive fluorodeoxyglucose (FDG)-avid supraclavicular, mediastinal, hilar, and pulmonary lymph nodes, as well as faint reactive uptake in a left axillary node (Figure 3).

Surgery was subsequently consulted for biopsy of these lymph nodes on Day 36. Mediastinoscopic biopsy retrieved firm lymph nodes exhibiting noncaseating granulomas; special stains for fungus and acid-fast bacilli were negative, establishing sarcoidosis.

Follow-up testing and methotrexate initiation

**Figure 4:**
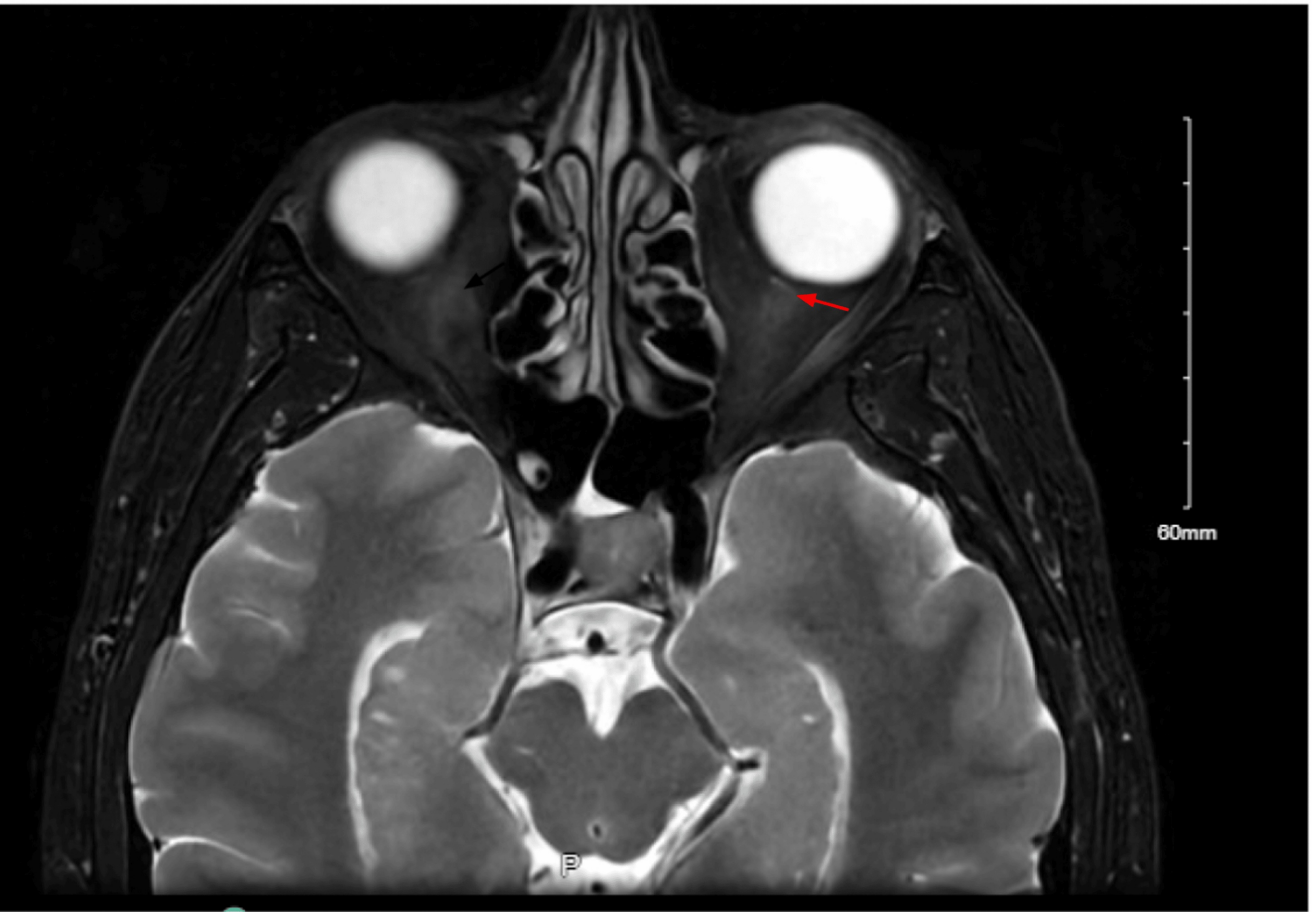
Follow-up T1 fat-saturated orbital MRI illustrating moderate resolution of optic nerve enhancement (red arrow) after corticosteroid therapy.

After completing his oral steroid taper, a follow-up orbital MRI on Day 41 showed significant resolution of optic nerve enhancement (Figure 4). The patient subsequently transitioned off oral corticosteroids and began weekly methotrexate (escalated to 15 mg) with folate rescue and vitamin D supplementation. By two months post-presentation, visual acuity had stabilized at 20/40 OS, color vision remained intact, and repeat pulmonary function testing, including spirometry, lung volumes, diffusion capacity, and six-minute walk testing, demonstrated preserved respiratory function with moderate exertional dyspnea.

Long-term follow-up

Over the ensuing year, serial MRI scans showed no recurrent optic nerve enhancement, and the patient reported sustained visual stability. Periodic pulmonary assessments confirmed stable lymphadenopathy without progressive parenchymal disease. Methotrexate was continued as a steroid-sparing agent, with ongoing monitoring for toxicity and disease relapse.

## Discussion

This case report describes a 43-year-old man whose isolated, unilateral painless optic neuritis proved to be the initial manifestation of systemic neurosarcoidosis. While facial nerve palsy is the most commonly reported cranial neuropathy in neurosarcoidosis, optic neuritis is far less frequent and may present without systemic symptoms. Although pulmonary involvement was discovered, the neurological presentation of optic neuritis was isolated (no meningitis, no other cranial neuropathies, and no parenchymal CNS lesions), justifying the term “isolated optic neuritis as the presenting feature.” The temporal sequence, from blurred vision and optic disc swelling to targeted imaging, cerebrospinal fluid analysis, and high-dose steroid therapy, highlights the importance of early neuro-ophthalmic evaluation.

Discovery of thoracic lymphadenopathy on chest imaging prompted PET-CT and tissue biopsy, confirming noncaseating granulomas and securing the diagnosis. High-dose corticosteroids provided rapid visual recovery, while methotrexate served as an effective long-term immunosuppressive strategy to maintain remission and prevent relapse. Early transition to steroid-sparing therapy helped reduce the risk of corticosteroid-associated toxicity while sustaining disease control.

This case aligns with rare presentations noted in the literature. Facial nerve palsy is more commonly reported as the predominant cranial neuropathy in neurosarcoidosis [5], whereas optic neuritis, as seen here, is less frequent but can lead to severe visual impairment if untreated [7]. The rapid visual recovery following high-dose corticosteroids and the sustained remission with methotrexate are consistent with findings from previous studies, which emphasize early intervention [6]. Unlike some cases where systemic symptoms precede neurological involvement [5], this patient lacked initial systemic signs, underscoring the need for comprehensive imaging and biopsy. Comparative studies, such as Zajicek et al. [8], highlight the variability in neurosarcoidosis presentations and the efficacy of steroid-sparing agents like methotrexate in preventing relapse, supporting the long-term management approach in this case.

Multidisciplinary collaboration between neurology, pulmonology, and rheumatology, coupled with serial MRI and pulmonary function monitoring, is essential to optimize outcomes, preserve vision, and mitigate treatment-related toxicity in neurosarcoidosis. Overall, this case accentuates the need to maintain a high index of suspicion for systemic sarcoidosis in patients presenting with isolated optic neuritis, and it reinforces the importance of timely imaging, biopsy, and coordinated care.

## Conclusions

Neurosarcoidosis should be considered in the differential diagnosis of optic neuritis, particularly when vision loss is severe, atypical, or unresponsive to standard therapies, and especially when systemic findings such as lymphadenopathy are present. This rare case demonstrates the importance of a thorough, stepwise evaluation, including neuroimaging, CSF analysis, and systemic workup with tissue biopsy, to establish a definitive diagnosis. Early initiation of high-dose corticosteroids can result in dramatic visual recovery, while long-term immunosuppression with agents like methotrexate is critical to maintain remission and prevent relapse. Ultimately, a multidisciplinary, patient-centered approach and vigilant follow-up are key to optimizing visual and systemic outcomes in neurosarcoidosis.
